# Outcome devaluation by specific satiety disrupts sensory-specific Pavlovian-to-instrumental transfer

**DOI:** 10.3389/fnbeh.2022.983480

**Published:** 2022-11-09

**Authors:** Marios C. Panayi, Simon Killcross

**Affiliations:** ^1^School of Psychology, University of New South Wales, Sydney, NSW, Australia; ^2^National Institute on Drug Abuse Intramural Research Program, Baltimore, MD, United States

**Keywords:** Pavlovian, instrumental, transfer, specific satiety, devaluation, habituation, stimulus, motivation

## Abstract

Reward predictive cues can selectively motivate instrumental behaviors that predict the same rewarding outcomes, an effect known as specific Pavlovian-to-instrumental transfer (PIT). This selective effect is thought to be mediated by a representation of the sensory specific properties of an outcome, that has become associated with both the Pavlovian cue and the instrumental response during initial learning. Specific satiety is a common method of outcome devaluation that reduces an outcome's value but might also lead to the habituation of the outcome's sensory properties. Previous research has demonstrated that specific PIT is insensitive to changes in specific outcome value following taste aversion devaluation, as well as general satiety manipulations, and therefore specific satiety should not disrupt specific PIT by reducing outcome value. The present rodent experiments used a specific satiety devaluation procedure immediately prior to a specific PIT test to show that habituation of these outcome specific sensory representations can disrupt its efficacy as a stimulus and abolish the specific PIT effect. Experiment 1 employed a two-lever choice test to show that a non-devalued stimulus supports specific PIT, whereas a devalued stimulus abolished the specific PIT effect. Experiment 2 replicated this procedure while controlling for response competition by using a single-lever test to confirm that a devalued stimulus abolishes the specific PIT effect. These findings demonstrate that specific satiety can disrupt the ability of an outcome specific representation to support specific PIT. Given previous findings that specific PIT is insensitive to changes in outcome value by general satiety and taste aversion devaluation, this suggests that specific satiety devaluation might disrupt the use of sensory specific outcome representations to guide behavior *via* a mechanism that is independent of the outcome's current value.

## Introduction

During learning in Pavlovian and instrumental conditioning procedures, outcome representations often form a complex part of the associative structure that is established (Rescorla, 1988; Hall, 2002; Urcuioli, 2005; Delamater and Oakeshott, 2007). For example, in a Pavlovian learning task, a rat learning that an auditory tone cue predicts sucrose reward (Stimulus-Outcome; S-O) will often represent multiple aspects of this outcome event such as its flavor and texture (i.e., unique sensory properties), spatial location, timing, motivational value etc. Similarly, during instrumental conditioning, i.e., when learning lever press response for a sucrose reward (Response-Outcome; R-O), these multiple aspects of the outcome can also form part of the associative relationship with the response (Colwill and Motzkin, 1994; Balleine and Killcross, 2006; Delamater and Holland, 2008).

Experimental research has focused mostly on the distinction between the unique sensory-specific properties vs. the general-motivational properties of outcome representations (Konorski, 1967; Dickinson and Dearing, 1979), in particular, using Pavlovian-to-instrumental transfer (PIT) procedures. In a typical rodent full PIT procedure (Corbit et al., 2007; Cartoni et al., 2016), rats are first trained on three unique stimulus-outcome relationships, e.g., S_1_-O_1_/ S_2_-O_2_/S_3_-O_3_, and independently trained on two unique lever response-outcome relationships, e.g. R_1_-O_1_/R_2_-O_2_. Finally, the stimuli (S_1_/S_2_) are presented in the presence of the instrumental responses (R_1_/R_2_) for the first time in a PIT transfer test conducted in extinction. The general PIT effect describes the ability for a Pavlovian stimulus to increase responding on an independently trained instrumental response i.e., S_3_ will enhance responding on both R_1_ and R_2_. This PIT effect differentially biases responding when both the stimulus and response were trained with the same outcome i.e., S_1_ will preferentially enhance R_1_, and S_2_ will preferentially enhance R_2_. These distinct outcome specific and general PIT effects are strong evidence that the sensory-specific properties of the outcome (i.e., its identity) are an independent part of the associations formed during the initial Pavlovian and instrumental training.

Additional support for this distinction between the sensory-specific and general properties of outcomes in Pavlovian and instrumental learning, as well as PIT, comes from a growing body of neural evidence. For example, lesions or functional inactivation of the central amygdala (CeA), nucleus accumbens core (NAcc core), and ventral tegmental area disrupt general PIT while leaving specific PIT intact (Corbit et al., 2007), whereas targeting the basolateral amygdala (BLA), nucleus accumbens shell (NAcc shell), mediodorsal thalamus (MD), ventral pallidum, as well as frontal region such medial and lateral orbitofrontal cortex (mOFC, lOFC), selectively abolishes specific but not general PIT (Blundell et al., 2001; Corbit et al., 2001, 2003, 2007; Holland and Gallagher, 2003; Corbit and Balleine, 2005, 2011; Balleine and Killcross, 2006; Ostlund and Balleine, 2007a; Leung and Balleine, 2015; Balleine et al., 2011; Leung and Balleine, 2013; Ostlund and Balleine, 2008; Lichtenberg et al., 2017; Bradfield et al., 2018; Panayi and Killcross, 2018; Sias et al., 2021). While some of these neural pathways also underpin more general processes of Pavlovian and instrumental learning about the sensory specific and general properties of outcomes, there is compelling evidence to suggest neural processes that are unique to PIT. For example, successful expression of specific PIT uniquely depends upon the trafficking of delta-opioid receptors on cholinergic interneurons in the NAcc shell (but not NAcc core) during initial Pavlovian conditioning, which in turn modulate dopamine D1 (but not D2) receptor activity that is necessary for the learning and expression of specific PIT (Laurent et al., 2012, 2014; Bertran-Gonzalez et al., 2013).

The associative account of the specific PIT effect is that during the transfer test the Pavlovian stimulus will activate a representation of the expected outcome, including its sensory properties e.g., S_1_-S_O1_. This in turn will activate the associated instrumental response e.g., R_1_, either by a backwards R_1_- S_O1_ association, or by a forwards S_O1_-R_1_ association where the outcome has formed part of the discriminative stimulus for the response during acquisition (Trapold and Overmier, 1972; Colwill and Rescorla, 1988; Rescorla and Colwill, 1989; Rescorla, 1992; Colwill, 1994; for a discussion of this theoretical distinction see Ostlund and Balleine, 2007b; Gilroy et al., 2014). For simplicity, we will discuss the signaling properties of the outcome (S_O_) in this associative chain as S_1_-S_O1_-R_1_. Importantly, if the outcome is devalued by forming a taste aversion immediately before a PIT test, rats will show an intact specific PIT effect despite showing independent evidence of outcome specific devaluation on the underlying Pavlovian, instrumental, and consummatory responses (Colwill and Rescorla, 1988, 1990b; Rescorla, 1994; Holland, 2004). This suggests that the learned signaling properties of S_O_ can act independently of the current motivational value of the expected outcome (e.g., based on levels of hunger). A complementary piece of evidence for this dissociation is that reducing hunger (i.e., general satiety) abolishes the general, i.e., non-outcome specific, form of PIT but not specific PIT (Corbit et al., 2007; Lingawi et al., 2022; Sommer et al., 2022). Thus, specific PIT is argued to be unaffected by manipulating the current outcome value or general motivation for the outcome because these manipulations leave S_O_, the sensory properties of the expected outcome, intact.

A prediction of this account is that the specific PIT effect should be reduced by a manipulation that reduces the ability to associatively activate a representation of S_O_. Habituation by repeatedly presenting a stimulus has been shown to temporarily reduce the ability to associatively activate representations of the stimulus in a stimulus-specific manner (Wagner, 1981; Rankin et al., 2009; Lloyd et al., 2014). Indeed, during the course of standard instrumental training, McSweeney and Murphy (2009) review a large body of work which demonstrates that instrumental responding often declines within-session because repeated deliveries of the outcome leads to sensory specific habituation of S_O_ rather than a general loss of hunger or motivation (see also Epstein et al., 2009; Bouton et al., 2013). Therefore, habituation of S_O_ by repeated pre-exposure to that outcome should reduce the likelihood of associatively activating S_O_, thus impairing specific PIT for that outcome *via* an S-S_O_-R pathway. Notably, extensive pre-exposure to an outcome before a test is used to induce outcome specific satiety (e.g., 1 h of unlimited access enabling the subject to voluntarily pre-expose themselves to the reinforcer to the greatest extent possible), another common method of outcome devaluation (Panayi and Killcross, 2018). A potential confound caused by specific satiety is that it also reduces the incentive value of the outcome (Balleine and Dickinson, 1998) as well as generally satiating the animal, however both of these factors do not disrupt specific PIT (Colwill and Rescorla, 1988, 1990b; Rescorla, 1994; Holland, 2004; Corbit et al., 2007; Lingawi et al., 2022). Here we tested this hypothesis that specific satiety will disrupt specific PIT.

Two recent studies have tested the effects of specific satiety on specific PIT in rodents and both reported that, similar to devaluation with taste aversion, specific satiety did not abolish specific PIT (Lingawi et al., 2022; Sommer et al., 2022). However, both studies employed two-lever choice tests, i.e., both the devalued and non-devalued lever were present during the PIT test, which complicates the interpretation of these findings. Differences in PIT can be the result of different baseline levels of responding on the devalued and non-devalued lever (Rescorla, 1994; Holland, 2004; Holmes et al., 2010; Cartoni et al., 2016), or competition between responses (Laurent and Balleine, 2015; Lovibond et al., 2015). For example, both studies report significantly lower responding on the devalued than the non-devalued lever during the baseline periods. Indeed, the specific PIT effect in the presence of a devalued stimulus reported by Lingawi et al. (Figure 3I in Lingawi et al., 2022) reflects a small but significant suppression in responding on the different outcome lever, but no evidence of elevated responding on the same outcome lever i.e., no specific PIT. The findings of (Sommer et al., 2022) show a small but significant specific PIT effect in the presence of the devalued stimulus but not the non-devalued stimulus (Figure 1D in Sommer et al., 2022). Therefore, the findings of these studies do not unambiguously disconfirm our prediction that specific satiety will disrupt specific PIT.

The present experiments were conducted prior to these two recent reports and were not specifically designed in response to these findings; however, the design of our PIT procedure overcomes some of the issues related to the two lever choice tests described above. Experiment 1 employed a two-lever choice test but preceded the test with a longer instrumental extinction period that eliminated difference in baseline responding on the levers. Experiment 2 replicated this design but employed a one-lever specific PIT test. Our findings supported our hypothesis such that specific satiety devaluation selectively abolished specific PIT for the devalued outcome but not for the non-devalued outcome.

## Materials and methods

### Animals

Rats were housed four per cage in ventilated Plexiglass cages in a temperature regulated (22 ± 1¬°C) and light regulated (12 h light/dark cycle, lights on at 7:00 AM) colony room. At least 1 week prior to behavioral testing, feeding was restricted to ensure that weight was ~95% of *ad libitum* feeding weight, and never dropped below 85% (achieved by providing 15 g lab chow per rat per day, and monitoring weight at least twice a week). All animal research was carried out in accordance with the National Institute of Health Guide for the Care and Use of Laboratories Animals (NIH publications No. 80-23, revised 1996) and approved by the University of New South Wales Animal Care and Ethics Committee. Subjects were and 32 male Wistar rats (BRC Laboratory Animal Service, University of Adelaide, South Australia, Australia) approximately 4 months old (Experiment 1, *N* = 16, weighing between 326 and 475 g, M = 386.0 g; Experiment 2, *N* = 16, weighing between 300 and 435 g, M = 373.4 g).

### Apparatus

#### Test chambers

Behavioral testing was conducted in eight identical operant chambers (30.5 × 32.5 × 29.5 cm; Med Associates) individually housed within ventilated sound attenuating cabinets. Each chamber was fitted with a 3-W house light that was centrally located at the top of the left-hand wall. Food pellets could be delivered into a recessed magazine, centrally located at the bottom of the right-hand wall. Delivery of up to two separate liquid rewards *via* rubber tubing into the magazine was achieved using peristaltic pumps located above the testing chamber. The top of the magazine contained a white LED light that could serve as a visual stimulus. Access to the magazine was measured by infrared detectors at the mouth of the recess. Two retractable levers were located on either side of the magazine on the right-hand wall. A speaker located to the right of the house light could provide auditory stimuli to the chamber. In addition, a 5-Hz train of clicks produced by a heavy-duty relay placed outside the chamber at the back right corner of the cabinet was used as an auditory stimulus. The chambers were wiped down with ethanol (80% v/v) between each session. A computer equipped with Med-PC software (Med Associates Inc., St. Albans, VT, USA) was used to control the experimental procedures and record data. Throughout all stages of behavioral training in the test chambers, the house light and fan were always on.

#### Devaluation chambers

To provide individual access to reinforcers during the devaluation procedure, rats were individually placed into a clean mouse-sized home cage (33 x 18 x 14 cm clear Perspex cage with a wireframe top). Liquid reinforcers were presented in water bottles with a sipper tube. One day prior to the start of the devaluation period, all rats were exposed to the devaluation cages and given 30 mins of free access to home cage food and water to reduce novelty to the context and any potential neophobia to drinking from the water bottles.

#### Reinforcers

The reinforcers used were a single grain pellet (45 mg dustless precision grain-based pellets; Bio-serv, Frenchtown, NJ, USA), 20% w/v lemon flavored sucrose solution and 20% w/v peppermint flavored maltodextrin solution (Myopure, Petersham, NSW, Australia). Liquid reinforcers were flavored with either 0.4% v/v concentrated lemon juice (Berri, Melbourne, Victoria, Australia) or 0.2% v/v peppermint extract (Queen Fine Foods, Alderley, QLD, Australia) to provide unique sensory properties to each reinforcer. Liquids were delivered over a period of 0.33 s *via* a peristaltic pump corresponding to a volume of 0.2 mL. The volume and concentration of liquid reinforcers was chosen to match the calorific value of the corresponding grain pellet reward and have been found to elicit similar rates of Pavlovian and instrumental responding as a pellet reward in other experiments conducted in this lab. In all sessions involving liquids, the magazine was scrubbed with warm water and thoroughly dried between sessions to remove residual traces of the liquid reinforcer. To reduce neophobia to the reinforcers, 1 day prior to magazine training sessions all rats were pre-exposed to the reinforcers (10 g of pellets per rat and 25 ml of each liquid reinforcer per rat) in their home cage.

### Behavioral procedures

The behavioral procedures for Experiment 1 and Experiment 2 were identical except for the number of levers extended during the final PIT test session. Experiment 1 employed a two-lever choice test, whereas Experiment 2 employed a single-lever test procedure (described in detail below).

#### Magazine training

All rats received three sessions of magazine training, one for each reinforcer with the following parameters: reward delivery was on a random time 60 s schedule (RT60s) for 16 rewards. Sessions occurred on three consecutive days with the order of reward identity counterbalanced between rats.

#### Lever training

Following magazine training, all rats were given 2 days of lever training on a continuous reinforcement schedule (each lever press was rewarded) with the same parameters as the instrumental training sessions described below.

#### Acquisition training

On each day all rats received either a single Pavlovian training session, or two instrumental training sessions. The order of Pavlovian and instrumental sessions alternated each day.

#### Pavlovian training

All rats received a total of 6 days of Pavlovian training. Pavlovian training sessions consisted of 3 stimuli (CS), a tone (2,800 Hz, 80 dB), white noise (78 dB), and a clicker (5 Hz). There were 4 presentations of each CS (i.e., a total of 12 cues presented within a session) each lasting 2 min with a variable ITI of 300 s. Reward was delivered throughout the cue period on a RT 30 s schedule. Each cue was paired with a unique outcome (grain pellet, lemon sucrose, and peppermint maltodextrin) and the identity of the cue-outcome relationship remained constant for each rat (counterbalanced between rats).

#### Instrumental training

All rats received a total of 6 days of instrumental training. Instrumental training involved two sessions per day, separated by at least 1 h. During the session a single lever was extended, and lever pressing was rewarded with a unique liquid outcome, either lemon sucrose or peppermint maltodextrin. During the second instrumental session of the day, a different lever was extended, and lever pressing was rewarded with the unique liquid outcome that was not paired with the previous lever. The identity of the lever outcome pairings was kept consistent within subjects and was counterbalanced between subjects. Training sessions lasted until a maximum of 20 rewards was earned or until 30 min had elapsed. On the first 2 days, reinforcement was delivered on a random ratio 5 schedule (RR5) such that on average a reward was delivered every 5 lever presses, followed by 4 days of RR10.

#### Devaluation

Satiety devaluation was achieved by providing rats with 1 h of free access to one of the liquid reinforcers in the devaluation chamber. At the end of the 1-h period, rats were removed from the devaluation chamber and put back in their home cage, and immediately transferred to the test chambers.

In experiment 1, devaluation occurred on two consecutive days with a different liquid reinforcer. In experiment 2, devaluation occurred on two consecutive days with the same reinforcer to assess the effect of devaluation of the same outcome on both levers in separate sessions. Following 2 further days of Pavlovian and instrumental retraining, the alternative liquid reinforcer was then devalued for 2 days. This resulted in both liquid reinforcers being devalued and tested with each lever.

#### Extinction and PIT test

The PIT test started with lever extinction, both levers were extended in Experiment 1, and only a single lever was extended in Experiment 2. Lever pressing had no programmed consequences throughout the entire session. After 10 min, the CSs were presented (duration 2 min) with a fixed 2 min inter-stimulus interval. Each CS was presented three times (a total of 9 CS presentations) and the order of CS presentation was randomized. In Experiment 1 involving two levers, the PIT test was repeated on the following day (i.e., once per devalued outcome). In Experiment 2 involving only a single lever test session, a second test was repeated on the following day with the lever that had yet to be tested. Order of lever presentation was counterbalanced. This pattern of two single lever tests was then repeated after 4 days of retraining on Pavlovian and instrumental sessions (i.e., a total of 4 PIT test sessions, once per devalued outcome-lever combination).

Starting the PIT test with extinction of the levers served multiple purposes. First, it reduced lever pressing behavior to a low baseline response rate, which allows for clearer demonstration of the potential rate-enhancing effect of CS presentations on lever pressing i.e., the PIT effect. Secondly, the extinction period served as an instrumental outcome devaluation test to confirm the efficacy of the specific satiety devaluation manipulation. Thirdly, it minimizes any differences in baseline rates of responding between the levers associated with the devalued and non-devalued outcomes. Differences in baselines can limit the interpretation and expression of any differences in the PIT effect on each lever.

### Data analysis

For the Pavlovian stage, a CS-PreCS elevation score was calculated by subtracting the rate of magazine entry during the 2 min immediately before each CS (PreCS) from the 2 min CS period. Lever pressing rates were analyzed for the instrumental stage. During the specific satiety devaluation stage, the amount of liquid reinforcer (in grams) consumed in 1 h was calculated. Both magazine entry and lever pressing rates were calculated for the PIT test. During the extinction period, lever pressing was analyzed during the first 9 min in time blocks of 3 min (note that the last minute of the 10-min extinction period was part of the baseline period for the PIT; Supplementary Figure 1A). During the PIT test, both lever pressing and magazine entries were analyzed as elevation scores during the CS presentation above baseline. Here, because activity during the CS persisted for a short time after the CS ended, baseline responding was defined as responding in the 1 min before each stimulus (i.e., the last minute of the inter-stimulus-interval period). All response rates were calculated as responses per minute.

Data were analyzed using repeated measures ANOVAs with R statistical software (R Core Team, 2021), using the afex package (Singmann et al., 2022) implementation of the aov_4 function, with a multivariate model for all follow up tests (setting: emmeans_model = “multivariate”). Simple effects were used to explore significant main effects and interactions using the emmeans package (Lenth, 2022), with a Tukey method of familywise error-rate correction. Simple effects for the analysis of rates of acquisition were conducted using the linear component of planned orthogonal trend contrasts. Repeated measures *t*-tests were used for analyses with only two conditions.

During the PIT test, three analysis strategies were planned to look at the relationship between the expected outcomes of the Pavlovian stimuli and instrumental actions (Outcome Specificity: Same, Different, General). (1) The relationship between outcome specificity and whether the outcome of the instrumental lever was devalued or non-devalued (Lever Devaluation). (2) Whether the stimuli increased lever pressing above baseline in each condition, indicating some form of PIT transfer. (3) The relationship between outcome specificity and whether the outcome of the Pavlovian stimuli was devalued or non-devalued (Stimulus Devaluation). Note that analysis options (1) and (3) do not change the underlying data (and are identical when only a single lever is present as in Experiment 2). Instead, both analyses were conducted to aid comparisons with earlier studies and provide a complete exploration of the complex experimental design (for a detailed discussion, see Supplementary Figure 2).

#### Exclusions

Two rats were excluded from Experiment 2 based on a substantial response bias to one cue. Responding was over 4x higher to one CS suggesting substantial cue or outcome preference.

## Results

### Experiment 1. Specific satiety abolishes specific PIT in a two-lever choice test

**Figure 1:**
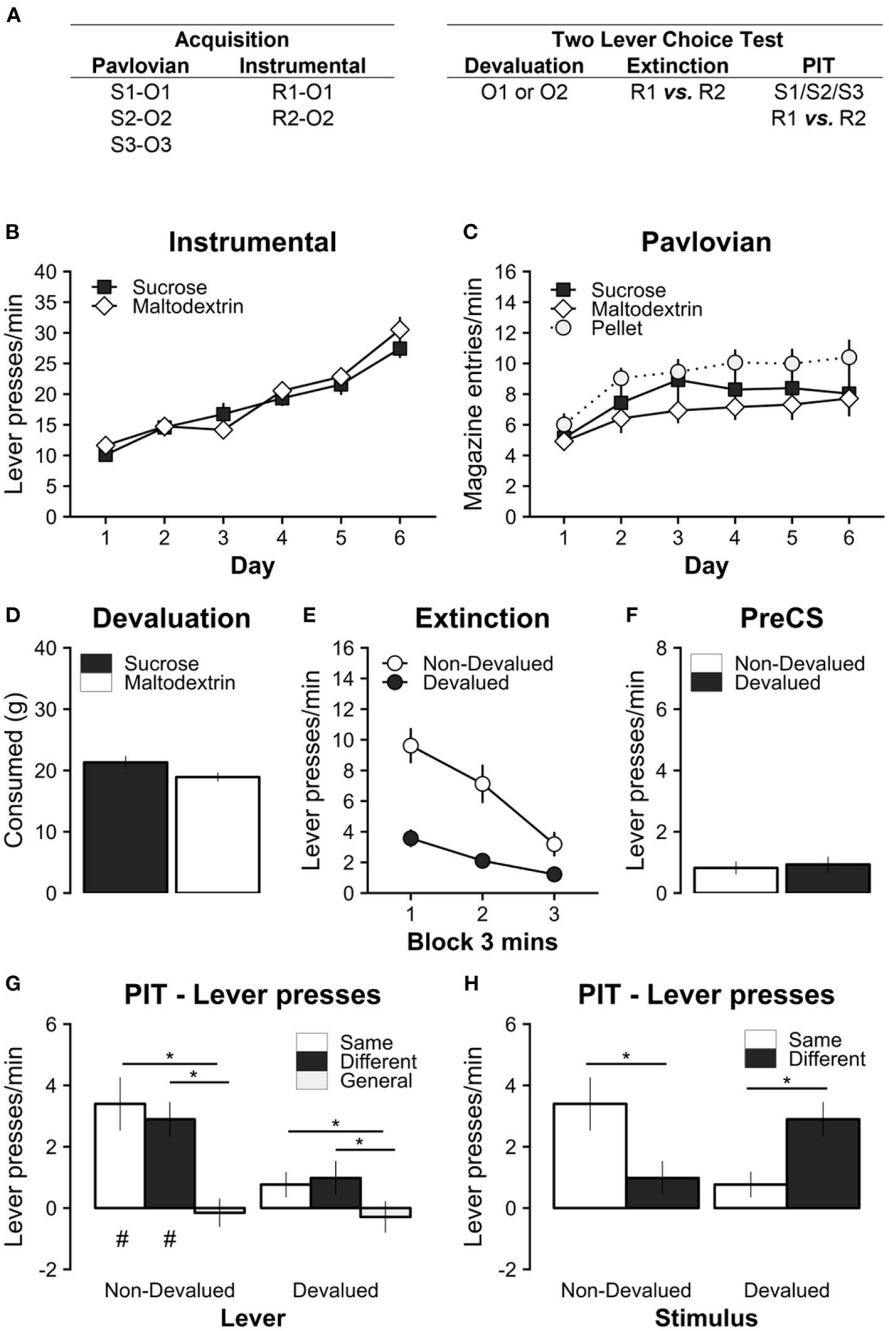
Experiment 1 tested the effect of outcome devaluation by sensory-specific satiety on specific PIT in a two-lever choice test. **(A)** Experimental design; S1/S2/S3: clicker, tone, and noise stimuli (counterbalanced); R1/R2: left and right lever press actions (counterbalanced); O1/O2: sucrose and maltodextrin liquid reinforcer outcomes (counterbalanced); O3: pellet reinforcer outcome. **(B)** All rats learned to perform the left and right lever responses for the sucrose and maltodextrin liquid reinforcers. **(C)** All rats learned that the Pavlovian stimuli uniquely predicted the sucrose, maltodextrin, and pellet outcomes. **(D)** During the 1-h specific satiety devaluation session, rats consumed a substantial amount of the reinforcer, but consumed slightly more when the reinforcer was sucrose. **(E)** Outcome devaluation successfully reduced the rate of lever pressing on the lever associated with the devalued outcome during the extinction period with both levers present. **(F)** Baseline responding during the PIT test was similar on the devalued and non-devalued levers. **(G)** Outcome devaluation abolished specific PIT when separating responses on the non-devalued (Left) and devalued (Right) levers. **(H)** Outcome devaluation reversed specific PIT when separating responses during the non-devalued (Left) and devalued (Right) stimuli. Note that the same data are presented in **(G)** and **(H)**. * Significant simple effects. p < 0.05. # Significant responding above baseline, p < 0.05. Full analysis details in main text. Data are presented as mean ± SEM.

**Figure 2:**
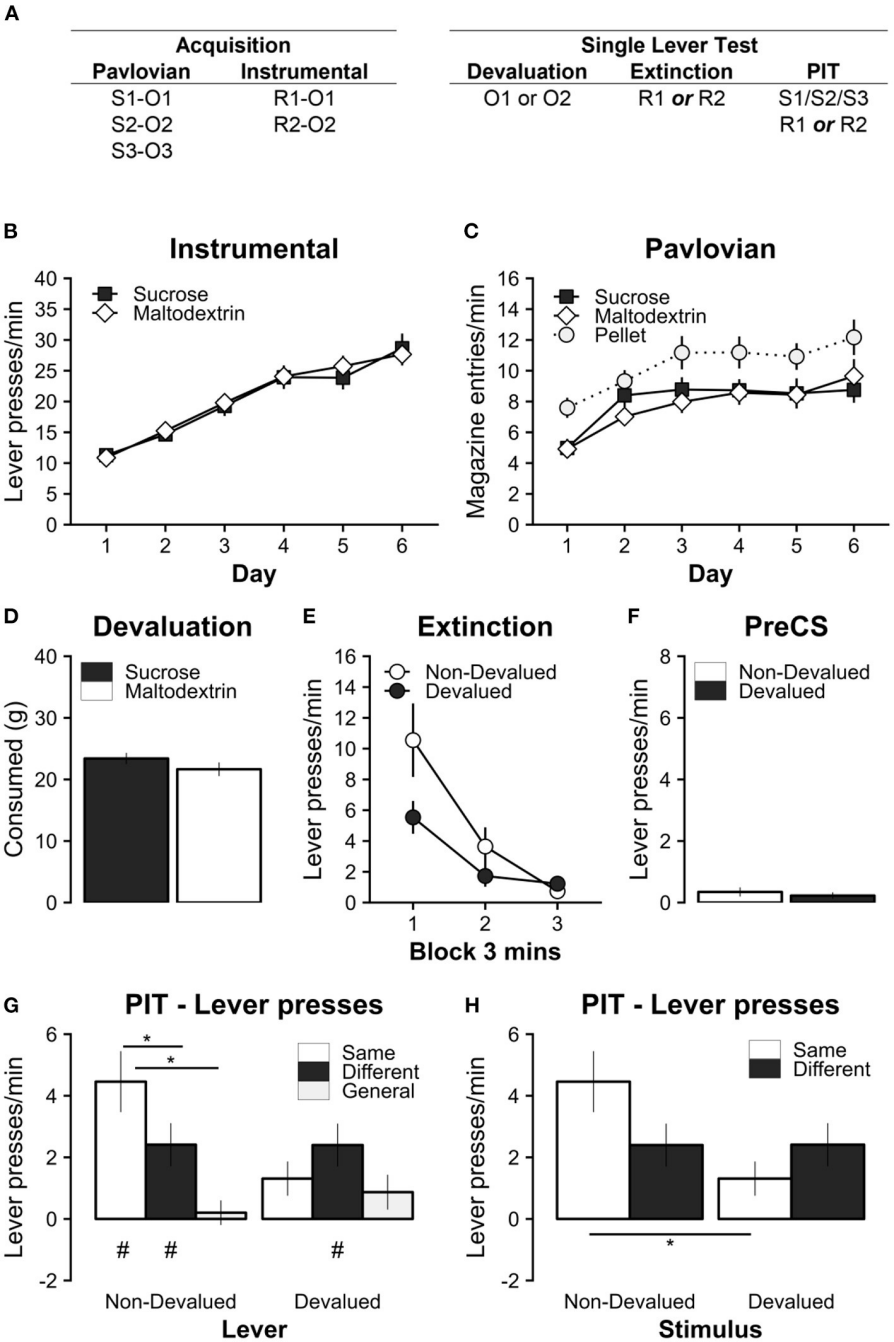
Experiment 2 tested the effect of outcome devaluation by sensory-specific satiety on specific PIT in a single-lever test. **(A)** Experimental design; S1/S2/S3: clicker, tone, and noise stimuli (counterbalanced); R1/R2: left and right lever press actions (counterbalanced); O1/O2: sucrose and maltodextrin liquid reinforcer outcomes (counterbalanced); O3: pellet reinforcer outcome. **(B)** All rats learned to perform the left and right lever responses for the sucrose and maltodextrin liquid reinforcers. **(C)** All rats learned that the Pavlovian stimuli uniquely predicted the sucrose, maltodextrin, and pellet outcomes. **(D)** During the 1-hour specific satiety devaluation session, rats consumed a substantial amount of the reinforcer, but consumed slightly more when the reinforcer was sucrose. **(E)** Outcome devaluation successfully reduced the rate of lever pressing on the lever associated with the devalued outcome during the extinction period with only a single lever present in each test. **(F)** Baseline responding during the PIT test was similar on the devalued and non-devalued levers. **(G)** Outcome devaluation abolished specific PIT when separating responses on the non-devalued (Left) and devalued (Right) levers. **(H)** Outcome devaluation reversed specific PIT when separating responses during the non-devalued (Left) and devalued (Right) stimuli. Note that the same data are presented in **(G)** and **(H)**. * Significant simple effects. *p* < 0.05. # Significant responding above baseline, *p* < 0.05. Full analysis details in main text. Data are presented as mean ± SEM.

Experiment 1 assessed the impact of specific satiety on the ability of a Pavlovian CS to invigorate actions that produce the same outcome (Figure 1A). A full transfer paradigm was used (Cartoni et al., 2016). Rats (*n* = 16) were first trained with three unique Pavlovian CS-outcome relationships (i.e., S1-O1, S2-O2, S3-O3) and two unique instrumental lever response-outcome relationships (i.e., R1-O1, R2-O2) on alternating days (see Figures 1A, 2A). Next, one of the instrumental outcomes (O1 or O2) was devalued by specific satiety immediately before a two-lever PIT choice test. The PIT test started with the presentation of both levers and an extinction period to reduce baseline lever pressing before CSs were presented (also in extinction) to examine their impact on instrumental lever pressing. O1 and O2 were always liquid reinforcers (sucrose and maltodextrin, counterbalanced), and O3 was always grain pellets. This ensured that devaluation of O1 or O2 was not confounded by any potential asymmetric effects when comparing liquid and solid food reinforcers.

During the PIT test there were three possible relationships between CSs and instrumental responses (1) Same: Pavlovian cues that predicted the same instrumental outcome (i.e. S1/R1, S2/R2), (2) Different Pavlovian cues that predicted a different instrumental outcome (i.e. S2/R1, S1/R2), and (3) General: the Pavlovian cue that predicted an outcome that was never an instrumental outcome (S3/R1, S3/R2). This test was repeated once following satiety devaluation with the instrumental outcome that was not presented before the first test.

#### Instrumental and Pavlovian conditioning

All rats successfully acquired instrumental lever responding (Figure 1A). Responding for both the sucrose and maltodextrin outcomes significantly increased over training days (main effect of Day, *F*(5, 75) = 63.39, *p* < 0.001; significant positive linear trend over Day, *t*(15) = 11.74, *p* < 0.001), and at comparable rates for both rewards (no main effect of Reward, *F*(1, 15) = 0.36, *p* = 0.559; or Day^*^Reward interaction, *F*(5, 75) = 1.47, *p* = 0.210).

All rats successfully acquired increased Pavlovian magazine responding during the CS (CS-PreCS elevation scores) for sucrose, maltodextrin, and pellets (Figure 1B). Magazine responding significantly increased over training days (main effect of Day, *F*(5, 75) = 9.94, *p* < 0.001; significant positive linear trend over Day *t*(15) = 4.18, *p* = 0.001), however overall responding was higher for pellets than for sucrose or maltodextrin (significant main effect of Reward *F*(2, 30) = 3.82, *p* = 0.033; but no significant Day^*^Reward interaction *F*(10, 150) = 0.46, *p* = 0.911). Simple main effects of Reward did not support this statistical difference after family-wise error rate correction (Maltodextrin vs. Pellet: *t*(15) = −2.51, *p* = 0.059, Sucrose vs. Pellet: *t*(15) = −1.71, *p* = 0.236, Maltodextrin vs. Sucrose: *t*(15) = −1.18, *p* = 0.483). The slightly elevated rate of magazine approach for pellets is likely to be due to the nature of the consummatory response (i.e., drinking liquid vs. chewing pellets) which are conflated with anticipatory approach in these response data. Importantly, there were no significant differences in the rate of instrumental and Pavlovian acquisition of the to-be-devalued rewards i.e., sucrose and maltodextrin.

#### Outcome devaluation and extinction

During 1 h of free access to one of the instrumental outcomes, rats readily consumed both sucrose and maltodextrin (Figure 1D). Consumption was marginally greater for sucrose than maltodextrin (Sucrose vs. Maltodextrin, *t*(15) = 2.17, *p* = 0.046).

Immediately following this specific satiety manipulation, instrumental responding was extinguished with both levers present for 9 min (Figure 1E). This extinction test confirmed that the devaluation manipulation was successful as it selectively reduced lever pressing for devalued reward. Responding on the devalued lever was significantly reduced compared to the non-devalued lever (significant main effect of Devaluation *F*(1, 15) = 31.63, *p* < 0.001; main effect of Time *F*(2, 30) = 22.14, *p* < 0.001; and Devaluation^*^Time interaction *F*(2, 30) = 7.13, *p* = 0.003).

#### Specific PIT: Two lever choice test

The specific PIT test followed immediately after instrumental extinction. Baseline lever responding during the PreCS baseline period (1 min prior to stimulus presentation) was low and did not differ between non-devalued and devalued levers (Figure 1F; Non-Devalued vs. Devalued: *t*(15) = −0.39, *p* = 0.705). The absence of significant differences in baseline responding confirmed that it was appropriate to analyze PIT test responding during stimulus presentation as an elevation score (responding during stimulus–baseline) in subsequent analyses. In contrast, baseline responding on the devalued lever was reported as significantly lower than the non-devalued lever in previous studies testing the effect specific satiety on specific PIT (Lingawi et al., 2022; Sommer et al., 2022).

During the PIT test, the relationship between the expected outcomes of the Pavlovian stimuli and instrumental actions (Specific PIT: Same, Different, General) was first separated based on the devaluation status of the instrumental action (Lever Devaluation: Non-Devalued, Devalued; Figure 1G). Overall response levels in the presence of the cues, relative to baseline, were significantly higher on the non-devalued than the devalued lever (main effect of Lever Devaluation *F*(1, 15) = 15.85, *p* = 0.001), which reflects a persistent effect of instrumental devaluation. Overall responding did not differ between the same and different cues [main effect of Specific PIT, *F*(2, 30) = 9.11, *p* = 0.001; simple main effect of Same vs. Different, *t*(15) = 0.25, *p* = 0.966, *d* = 0.24, 95% CI (−2.35, 2.85)], but responding to both same and different cues was significantly elevated compared to responding for the general cue [Same vs. General, *t*(15) = 3.16, *p* = 0.017, *d* = 3.16, 95% CI (0.56, 5.76); Different vs. General, *t*(15) = 4.44, *p* = 0.001, *d* = 4.44, 95% CI (1.85, 7.05)]. While the pattern of results also suggests that the magnitude of the PIT effect was greater for the non-devalued than the devalued lever, this was not supported by a statistical interaction [Lever Devaluation^*^PIT interaction, *F*(2, 30) = 1.95, *p* = 0.160]. Therefore, sensory specific satiety abolished specific PIT on both the devalued and non-devalued lever. However, it should be noted that since this is a two-lever choice test, this analysis obscures the important effects of response competition between the levers within each trial (addressed below). There was also no evidence of any differences in magazine responding to the devalued, non-devalued, or general stimulus (main effect of Stimulus Devaluation *F*(2, 30) = 0.02, *p* = 0.980; Supplementary Figure 1B).

We performed a second analysis on these data to determine whether there was any evidence of PIT, i.e., the ability for the Pavlovian CS to invigorate instrumental responding, for each stimulus-lever-outcome relationship. This was done by testing whether responding was above baseline i.e., are baseline subtracted scores significantly above zero? This was tested using the individual parameter effect estimates from the ANOVA model above (Figure 1G). This analysis suggested that responding was significantly elevated above baseline on the non-devalued lever for both the same and different stimulus conditions [Non-Devalued: Same, *t*(15) = 3.89, *p* = 0.009, *d* = 3.9, 95% CI (0.87, 6.92); Different, *t*(15) = 5.17, *p* = 0.001, *d* = 5.18, 95% CI (2.14, 8.19); General, *t*(15) = −0.34, *p* > 0.999, *d* = −0.35, 95% CI (−3.37, 2.68)], but not on the devalued lever [Devalued: Same, *t*(15) = 1.85, *p* = 0.408, *d* = 1.86, 95% CI (−1.16, 4.89); Different, *t*(15) = 1.77, *p* = 0.460, *d* = 1.77, 95% CI (−1.26, 4.8); General, *t*(15) = −0.57, *p* = 0.994, *d* = −0.56, 95% CI (−3.6, 2.45)].

Another approach to analyzing these data is to compare the relationship between the expected outcomes of the Pavlovian stimuli and instrumental actions (Specific PIT: Same, Different) with the devaluation status of the Pavlovian cues (Stimulus Devaluation: Non-Devalued, Devalued; Figure 1H). This provides a different way of visualizing these data, consistent with earlier studies, that directly compares the two-levers present during each trial (Supplementary Figure 2). Again, the pattern of responding suggests that specific satiety devaluation abolished specific PIT. The non-devalued stimulus elicited a significantly greater response on the same than the different lever [Non-Devalued: Same vs. Different, *t*(15) = 3.03, *p* = 0.009, *d* = 3.03, 95% CI (0.89, 5.16)], whereas the devalued stimulus elicited the opposite pattern of responding [Devalued: Same vs. Different, *t*(15) = 3.03, *p* = 0.009, *d* = 3.03, 95% CI (0.89, 5.16); significant Stimulus Devaluation^*^Specific PIT interaction, *F*(1, 15) = 28.93, *p* < 0.001; no main effect of Stimulus Devaluation, *F*(1, 15) = 0.18, *p* = 0.674, or Specific PIT, *F*(1, 15) = 0.06, *p* = 0.807]. This suggests that devaluation not only abolished but reversed the specific PIT effect. However, it is important to interpret this finding with caution because it is possible that the reversal of the specific PIT effect is being driven by an overall reduction in approaching the devalued lever, and response competition with the non-devalued lever. Surprisingly, both the devalued and non-devalued stimulus elicited similar levels of responding on the non-devalued lever. This suggests that the devalued stimulus conferred some form of general transfer effect (or a counterfactual association e.g., Laurent and Balleine, 2015), in contrast to the general stimulus which did not increase responding on either lever.

Finally, we also included session number into the analysis to confirm that these effects were consistent across repeated test sessions (i.e., Figure 1H split by session; data not shown). While there was an overall reduction in total responding over repeated testing [main effect of Session *F*(1, 15) = 9.45, *p* = 0.008], there were no interactions between test session and any other factors [Session^*^Stimulus Devaluation, *F*(1, 15) = 0.05, *p* = 0.822; Session^*^Specific PIT *F*(1, 15) = 0.12, *p* = 0.736; Session^*^Stimulus Devaluation^*^Specific PIT *F*(1, 15) = 0.42, *p* = 0.529].

### Experiment 2. Specific satiety abolishes specific PIT in a single lever test

The previous experiment revealed that the expression of specific PIT not only abolished but reversed by satiety devaluation in a two-lever choice test. To account for the potential effect of response competition between the devalued and non-devalued levers, Experiment 2 used an identical procedure but with only a single lever made available during the PIT test session (Figure 2A). Rats were first given two tests on one lever (e.g., R1) with a different outcome devalued before each session (i.e., O1 and O2), and then tested twice on the other lever (e.g., R2, devaluing O1 and O2). Rats were given brief reacquisition training on the Pavlovian and instrumental contingencies after the first two PIT sessions to minimize the effects of testing in extinction.

#### Instrumental and Pavlovian conditioning

As before, all rats successfully acquired instrumental lever responding (Figure 2B). Responding for both sucrose and maltodextrin outcomes significantly increased over training days [main effect of Day, *F*(5, 65) = 46.35, *p* < 0.001; significant positive linear trend over Day, *t*(13) = 9.64, *p* < 0.001], and at comparable rates for both rewards {no main effect of Reward, [*F*(1, 13) = 0.02, *p* = 0.888], or Day^*^Reward interaction, *F*(5, 65) = 0.41, *p* = 0.83).

All rats also successfully acquired robust Pavlovian magazine responding during the CS (CS-PreCS elevation scores) for sucrose, maltodextrin, and pellets (Figure 2C). Magazine responding for sucrose, maltodextrin, and pellets significantly increased over training days [main effect of Day, *F*(5, 105) = 12.79, *p* < 0.001; significant positive linear trend over Day, *t*(21) = 4.44, *p* < 0.001], however overall responding was higher for pellets than for sucrose or maltodextrin [significant main effect of Reward, *F*(2, 42) = 5.66, *p* = 0.007, but no significant interaction Day^*^Reward interaction, *F*(10, 210) = 0.69, *p* = 0.73]. Simple main effects of Reward revealed that responding for pellets was significantly higher than for sucrose or maltodextrin [Maltodextrin vs. Pellet, *t*(21) = −2.88, *p* = 0.024; Sucrose vs. Pellet, *t*(21) = −2.46, *p* = 0.056], but no significant difference between maltodextrin and sucrose [Maltodextrin vs. Sucrose, *t*(21) = −0.39, *p* = 0.920]. This replicates the trend that was observed in Experiment 1 and suggests that the nature of magazine responding for the pellet reward was different to the two liquid reinforcers. However, once again, there were no significant differences in the rate of instrumental and Pavlovian acquisition of the relevant to-be-devalued rewards i.e., sucrose and maltodextrin.

#### Outcome devaluation and extinction

After 1 h of free consumption prior to each test session, consumption was marginally greater for sucrose than maltodextrin [Sucrose vs. Maltodextrin, *t*(13) = 2.75, *p* = 0.017; Figure 2D]. However, ignoring outcome identity, total consumption of liquids did not differ across the 4 days of testing [main effect of Test Number, *F*(3, 39) = 2.52, *p* = 0.072].

Immediately following the specific satiety manipulation, rats were given 9 min of instrumental extinction with only a single lever present (Figure 2E). This extinction test confirmed that the devaluation manipulation was successful. Responding on the devalued lever was significantly reduced compared to the non-devalued lever in the first 3 min [Non-devalued vs. Devalued: Time Block 1, *t*(13) = −2.25, *p* = 0.042; Time Block 2, *t*(13) = −1.32, *p* = 0.210; Time Block 3, *t*(13) = 0.79, *p* = 0.444; supported by a significant Devaluation^*^Time interaction *F*(2, 26) = 4.03, *p* = 0.030; main effect of Time *F*(2, 26) = 25.20, *p* < 0.001; but not Devaluation, *F*(1, 13) = 3.77, *p* = 0.074]. It is noteworthy that the magnitude of the devaluation effect is not as profound as that observed in Experiment 1 (Figure 1E), which is likely due to greater sensitivity to differences in value and response competition in a simultaneous choice test.

#### Specific PIT: Single lever test

The specific PIT test followed immediately after instrumental extinction. Baseline lever responding during the PreCS baseline period (1 min prior to stimulus presentation) was low and did not differ between non-devalued and devalued levers [Figure 2F; Non-Devalued vs. Devalued: *t*(15) = −0.39, *p* = 0.705]. Like Experiment 1, the absence of significant differences in baseline responding confirmed that it was appropriate to analyze responding during stimulus presentation as an elevation score.

There was no evidence of differences in magazine responding during the PIT test (Supplementary Figure 1C). Magazine entries during the PIT test did not differ between lever devaluation or stimulus conditions [no main effect of Lever Devaluation, *F*(1, 13) = 0.81, *p* = 0.385; Stimulus Devaluation, *F*(2, 26) = 0.30, *p* = 0.740; or Lever Devaluation^*^Stimulus Devaluation interaction, *F*(2, 26) = 0.04, *p* = 0.959]. The lack of difference in magazine responses during stimulus presentation suggests that any differences in lever pressing during the PIT test are not being driven by differential response competition with the magazine response.

During the PIT test sessions with the non-devalued lever extended (Figure 2G, left), a significant outcome-specific PIT effect was observed such that lever pressing was greatest in the presence of the Same cue [Non-Devalued: Same vs. Different, *t*(13) = 2.85, *p* = 0.034, *d* = 2.85, 95% CI (0.21, 5.49); Non-Devalued: Same vs General, *t*(13) = 3.68, *p* = 0.007, *d* = 3.68, 95% CI (1.04, 6.32)] but there was no significant difference in lever responding to the Different and General cues [Non-Devalued: Different vs. General, *t*(13) = 2.39, *p* = 0.078, *d* = 2.39, 95% CI (−0.25, 5.04)]. In contrast, in test sessions with the devalued lever extended (Figure 2F, right), there were no significant differences in lever responding in the presence of any of the cues [Devalued: Same vs. Different, *t*(13) = −0.97, *p* = 0.605, *d* = −0.98, 95% CI (−3.61, 1.6); Devalued: Same vs. General, *t*(13) = 0.51, *p* = 0.867, *d* = 0.51, 95% CI (−2.13, 3.15); Devalued: Different vs. General, *t*(13) = 1.63, *p* = 0.267, *d* = 1.63, 95% CI (−1, 4.27)]. This differential expression of this specific PIT effect on the devalued and non-devalued levers was supported by a significant Lever Devaluation^*^Outcome-Specific interaction [*F*(2, 26) = 4.81, *p* = 0.017; a main effect of Outcome-Specific *F*(2, 26) = 6.18, *p* = 0.006; but no main effect of Lever Devaluation *F*(1, 13) = 3.93, *p* = 0.069]. Therefore, outcome devaluation by specific satiety selectively abolished specific PIT.

As before, we tested whether lever responding was elevated above baseline in each condition as another metric of PIT. Responding was significantly above baseline on the non-devalued lever for the Same and Different cue conditions [Non-Devalued: Same *t*(13) = 4.50, *p* = 0.004, *d* = 4.5, 95% CI (1.4, 7.59); Non-Devalued: Different *t*(13) = 3.44, *p* = 0.026, *d* = 3.44, 95% CI (0.34, 6.54); Non-Devalued: General *t*(13) = 0.51, *p* = 0.997, *d* = 0.5, 95% CI (−2.58, 3.61)], but only above baseline on the devalued lever for the Different cue [Devalued: Same *t*(13) = 2.35, *p* = 0.192, *d* = 2.35, 95% CI (−0.74, 5.45); Devalued: Different *t*(13) = 3.44, *p* = 0.026, *d* = 3.44, 95% CI (0.34, 6.54); Devalued: General *t*(13) = 1.54, *p* = 0.618, *d* = 1.54, 95% CI (−1.56, 4.63)]. This analysis confirms again that specific PIT was abolished by outcome devaluation with specific satiety. It also suggests that there was a form of general PIT on the devalued lever in the presence of the Different, but not to the General cue. Evidence of a PIT effect on the devalued lever also rules out the possibility that the strength of the devaluation on the instrumental lever prevented any form of PIT.

When considering whether the Pavlovian cue was devalued or non-devalued (Figure 2H), responding on the Same lever was significantly higher for the non-devalued than the devalued Pavlovian cue [Same: Non-Devalued vs Devalued, *t*(13) = 3.17, *p* = 0.007, *d* = 3.17, 95% CI (1.01, 5.33)], whereas responding on the different lever was almost identical for the Non-devalued and Devalued cue [Same: Non-Devalued vs. Devalued, *t*(13) = −0.01, *p* = 0.991, *d* = −0.01, 95% CI (−2.17, 2.15)]. Again, this pattern of differences in lever pressing was supported by a significant Pavlovian Devaluation^*^Outcome-Specific interaction [*F*(1, 13) = 5.87, *p* = 0.031; no significant main effect of Pavlovian Devaluation, *F*(1, 13) = 4.33, *p* = 0.058; or main effect of Outcome-Specific, *F*(1, 13) = 0.73, *p* = 0.409]. Therefore, specific PIT was abolished by specific satiety devaluation. This finding clarifies the findings of Experiment 1, and rules out alternative explanations of the effect being driven by differential baseline responding or response competition between the devalued and non-devalued levers.

Finally, we included session number into the analysis to confirm that these effects were consistent across repeated test sessions (i.e., Figure 2H split by session; data not shown). While there was an overall reduction in total responding over repeated testing [main effect of Session *F*(1, 13) = 42.56, *p* < 0.00]), there were no interactions between test session and any other factors [Session^*^Stimulus Devaluation, *F*(1, 13) = 1.62, *p* = 0.225; Session^*^Specific PIT *F*(1, 13) = 0.02, *p* = 0.888; Session^*^Stimulus Devaluation^*^Specific PIT *F*(1, 13) = 0.18, *p* = 0.678].

## Discussion

The present studies tested the prediction that specific satiety would abolish specific PIT. This was based on the hypothesis that outcome devaluation by sensory specific satiety leads to habituation of an outcome's sensory representation, which disrupts the capacity for this representation to support specific PIT. Experiment 1 tested this prediction with a two-lever choice-PIT test, whereas Experiment 2 used a single lever PIT test design to control for potential response competition between levers. Specific PIT was assessed using two criteria (1) greater responding on the same than different lever to demonstrate outcome specificity, and (2) responding on the same lever above baseline to demonstrate a facilitative PIT transfer effect on response levels. Therefore, the findings of both experiments supported our prediction that specific satiety devaluation would abolish specific PIT.

### Reports that specific satiety does not disrupt specific PIT

The present findings are in contrast to two recent reports of specific satiety leaving specific PIT intact, despite reducing the magnitude of effect (Lingawi et al., 2022; Sommer et al., 2022). Both studies used a two cue (S1-O1; S2-O2), two lever (R1-O1; R2-O2) design, where the outcomes were sucrose solution and grain pellets, a two-lever choice test, and a brief instrumental extinction period (between 2 and 6 min).

Sommer and colleagues report that a devalued stimulus is capable of supporting a significant specific PIT effect with responding on the same lever significantly above responding on the different lever and baseline (Figure 1D in Sommer et al., 2022). Surprisingly, the specific PIT effect they report was not as robust for the non-devalued stimulus, and baseline responding was significantly lower on the devalued than the non-devalued lever. Given the significant differences in baseline responding, it is unclear whether the magnitude of the specific PIT effect was reduced by specific satiety devaluation.

Lingawi and colleagues reported a significant reduction in the magnitude of the specific PIT effect for the devalued stimulus compared to the non-devalued stimulus (Figure 3I in Lingawi et al., 2022). However, the magnitude of the specific PIT effect was assessed by comparing baseline subtracted responding on the same and different levers. Close inspection of the data suggests that during the devalued stimulus responding on the same lever was not elevated above baseline, and instead the same-different effect is predominantly driven by a reduction in responding on the different lever (i.e., the non-devalued lever) relative to its baseline. Given that baseline responding was reported as significantly higher on the non-devalued lever, it is possible that an equivalently low response rate on both levers could produce this difference once different baseline response rates are subtracted. (Lingawi et al., 2022) tried to minimize differences in baseline responding by providing additional training on both levers with a common separate outcome (i.e., R1-O3; R2-O3), however this did not prevent robust differences in baseline responding. It is also possible that the different levels of baseline responding are the consequence of using the entire inter-stimulus period during the PIT test. In contrast, in the present experiments only the minute immediately before the stimulus was used as the baseline to remove the influence of any responding that persisted immediately after the previous stimulus ended.

The importance of having reasonably matched baseline responding has also been a point of focus for earlier work looking at the effect of taste aversion devaluation on specific PIT (Rescorla, 1994; Holland, 2004). This confound is particularly relevant when interpreting the magnitude of the specific PIT effect. In the present experiments, baseline lever responding was extinguished for significantly longer prior to the stimuli being presented, which successfully abolished significant differences in baseline levels that were used for calculating elevation scores. However, the present findings are qualitatively different from these two reports (Lingawi et al., 2022; Sommer et al., 2022) in that the magnitude of the specific PIT effect (i.e., comparing same vs. different) was not only abolished, but reversed for the devalued stimulus (supported statistically in Experiment 1, and numerically in Experiment 2). Therefore, it is unlikely that different baselines sufficiently account for these contrasting findings. Overall, the findings of the present study, and the results reported by (Lingawi et al., 2022) demonstrate a consistent sensory specific impact of satiety on the magnitude of the specific PIT effect.

Another consideration is that the mixed evidence of specific PIT observed in Experiment 1, unlike in Experiment 2, is the result of additional cognitive demands placed (e.g., conflicting response cues) on the animals during a two-lever choice test, which may also account for the differences observed with the reported findings. It is also possible that these conflicting findings are the result of methodological differences between each study, such as the specific rewards used or the relative amount of Pavlovian and instrumental training, which have been shown to influence the nature of the observed PIT effect (Holmes et al., 2010). Indeed, this is also evident in distinct nature of the basic specific PIT effect (i.e., in non-devalued sessions) reported by these studies. Specifically, while all procedures produced a robust and significant elevation in responding on the same lever, responding on the different lever was significantly elevated [present studies, (Panayi and Killcross, 2018)], significantly suppressed (Lingawi et al., 2022), or not different to baseline (Sommer et al., 2022). It is unclear whether these three different forms of specific PIT are differentially sensitive to specific satiety devaluation. However, it is also possible that the precise impact of specific satiety devaluation on specific PIT may be less clear without robust responding on the different lever (Supplementary Figure 2).

Surprisingly, both of these studies (Lingawi et al., 2022; Sommer et al., 2022) report a robust effect of specific satiety devaluation on magazine responding during the Pavlovian cues, whereas this effect was not found in the present experiments. This may reflect some differential sensitivity to Pavlovian and instrumental devaluation as a consequence of the specific training and testing parameters between procedures. Indeed, establishing different Pavlovian outcome expectancies (e.g., different outcome probabilities, uncertainty, and magnitude) have recently been shown modulate magazine-lever response competition at test in general PIT (Ostlund and Marshall, 2021). It is possible that similar factors may account for the differential impact of specific satiety devaluation on Pavlovian magazine responding between the present results and these recent reports (Lingawi et al., 2022; Sommer et al., 2022). Further work is needed to establish whether the magnitude of specific PIT is also influenced by these outcome expectancy properties.

### The effect of specific satiety on general PIT

A unique finding in the present results is that we observed robust elevation of responding on the Different lever. In Experiment 1, the devalued stimulus (but not the non-devalued stimulus) generated a robust increase in responding on the different lever i.e., both the devalued and non-devalued stimuli increased responding on the non-devalued lever (Figure 1G, Left). In Experiment 2, with only a single lever present at test, both devalued and non-devalued stimuli were capable of invigorating responding above baseline on the different lever at roughly similar levels. Elevated responding on the different lever has been predicted by theories of PIT, however responding on the different lever is usually reported to be no different to baseline responding (Cartoni et al., 2016; Lingawi et al., 2022; Sommer et al., 2022). Elevated responding on the different lever is hypothesized to be driven by a form of general PIT i.e., a non-specific energizing of the instrumental response driven by an association between the stimulus and the general motivational properties of the expected outcome (Balleine and Killcross, 2006; Corbit et al., 2007; Delamater and Oakeshott, 2007; Corbit and Balleine, 2015; Ostlund and Marshall, 2021).

Surprisingly, in both Experiment 1 and Experiment 2, the general stimulus (S_3_) did not drive any general PIT behavior. However, (Lingawi et al., 2022) have convincingly demonstrated that satiety manipulations with any outcome (either relevant or irrelevant to the Pavlovian or Instrumental conditions) abolishes general PIT. The absence of a general PIT effect with this stimulus in the present findings is therefore consistent with this result. However, we have previously reported an absence of general PIT with S_3_ in hungry/non-sated rats using the same single lever test design employed in Experiment 2 (appendix 1-Figure 1 in Panayi and Killcross, 2018). Therefore, despite the original intent of the experimental design, the specific parameters used do not generate general PIT with this stimulus S_3_. Furthermore, the elevated responding on the different lever (Experiment 2) might not be considered a general PIT effect either, as it was insensitive to non-specific satiety. However, we have not directly tested this assumption.

### The role of habituation processes in specific satiety

Our findings are consistent with evidence that specific satiety devaluation can lead to the habituation of an outcome's sensory representation (S_O_) and impact associated behaviors. Habituation describes the phenomenon where an initial response to a stimulus will decrease over repeated presentations of the stimulus (Thompson, 2009). Habituation is commonly used to describe sensory adaptation, for example, the perceived loss of flavor of the same food over the course of a meal. Indeed, sensory specific habituation is a mechanism that underlies how specific satiety changes food choice behavior (Epstein et al., 1992, 2009, 2010; Rolls, 2004). Beyond immediate consumption, sensory specific habituation has also been shown to be one key process driving the phenomenon of decreasing within-session instrumental lever pressing behavior in rats (McSweeney and Murphy, 2009). For example, the decline in within-session instrumental responding can be increased by introducing different or unexpected outcomes, even if these lead to increased effort or levels of satiety, which is consistent with dishabituation manipulations that disrupt habituation (Epstein et al., 2009; McSweeney and Murphy, 2009; Bouton et al., 2013). Therefore, there is empirical and theoretical support to suggest that habituation can play a role in how specific satiety impacts consummatory and appetitively motivated instrumental behaviors. A within-session decline in instrumental responding was also observed in the present experiments despite the relatively small number of outcomes (20) per session and short session length (10 min on average; Supplementary Figure 3).

Sensory specific habituation after satiety also appears to impact instrumental choice behavior over long periods of time. (Parkes et al., 2016) provide strong evidence for this showing that specific satiety devaluation in rats can affect instrumental choice behavior up to 2 h later, and even up to 5 h later when satiety and test occur in the same context. The long time course and context sensitivity of satiety devaluation on instrumental behavior suggests that behavioral control is being influenced by an associative recall process and retrieval interference mechanisms (Bouton, 1993). These findings are also consistent with accounts of long-term habituation where cues and contexts can prime representations of stimuli into working memory and modulate their ability to be recalled and influence learning and behavior (Wagner, 1981; Wagner and Brandon, 1989; Epstein et al., 2009; Robinson and Bonardi, 2015).

### Implications for devaluation

The effect of specific satiety reported here is important when contrasted with the consistent finding that outcome devaluation does not disrupt the expression of specific PIT when the outcome is devalued by taste aversion, often by pairing an outcome with LiCl induced illness (Colwill and Rescorla, 1990a; Rescorla, 1994; Holland, 2004). Indeed, these studies report that the size of the specific PIT effect is not reduced by taste aversion devaluation. In contrast, supporting our hypothesis, specific satiety devaluation either reversed (experiment 1), abolished (Experiment 2), or significantly reduces the size of the specific PIT effect (Lingawi et al., 2022).

Taste aversion and specific satiety methods of devaluation are often used interchangeably to probe an organism's ability to update behavior when the value of an outcome changes, and to establish learning about associations between cues and specific outcome identities and their neural substrates (Killcross and Blundell, 2002; Balleine et al., 2003). However, the present findings suggest that specific satiety might not engage the same associative mechanisms as taste aversion devaluation. These differences are of particular importance when using devaluation to probe the neural substrates of learning paradigms (Ostlund and Balleine, 2007c). However, it is important to note that the habituation mechanism being proposed is likely to be only one of multiple potential contributors to underlying a robust outcome devaluation effect experimentally.

### Limitations and future directions

An important limitation of the present study is that habituation was not directly manipulated independently of other satiety processes that may account for these findings. Instead, these alternative processes are ruled out by the results of other published experiments. It is therefore important to clearly identify each of these assumptions explicitly, their limitations, and to propose future experimental evidence required to overcome these limitations.

First is the issue of manipulating general levels of satiety. Is it the case that specific satiety reduces hunger and general motivation for appetitive outcomes, and therefore disrupts all forms of PIT, including specific PIT? It is reasonable to assume that specific satiety might generally disrupt specific PIT through a non-specific reduction in hunger and general motivation for appetitive outcomes. However, in the present experiments, the non-devalued stimulus and directly control for the impact of this general reduction in hunger. Furthermore, explicit general satiety manipulations have been shown to disrupt general but not specific PIT. This was first demonstrated by Corbit et al. (2007) (i.e., independently of whether rats were hungry or sated, responding to the Same but not the Different stimulus above baseline; a direct comparison of Same and Different conditions was not tested), and has been successfully replicated by multiple researchers (Lingawi et al., 2022; Sommer et al., 2022).

The second issue is that of manipulating specific outcome value, which we refer to as the hedonic value of the outcome (in contrast to general motivational value). Is it the case that during specific satiety, the hedonic value of the outcome is reduced, and specific PIT is sensitive to the current hedonic value of specific outcomes? While specific satiety does reduce hedonic value (Berridge, 1991), changing the hedonic value of an outcome using conditioned taste aversion has been shown to leave specific PIT effects intact (Colwill and Rescorla, 1988, 1990b; Rescorla, 1994; Holland, 2004). To what extent can the change in hedonic value induced by conditioned taste aversion be compared to specific satiety? Both specific satiety and taste aversion reduce positive hedonic responses to reinforcers (e.g., sucrose) in measures of taste reactivity, however only taste aversion (but not specific satiety) increases negative hedonic responses (Berridge et al., 1981; Berridge, 1991; Breslin et al., 1992). This suggests that both taste aversion and specific satiety reduce the hedonic value of an outcome. Furthermore, these changes in outcome value following specific satiety and taste aversion both depend upon incentive learning (Balleine, 1994; Balleine and Dickinson, 1998; Dickinson and Balleine, 2002). Taken together, it is therefore reasonable to conclude that the specific PIT effect is not sensitive to changes the current hedonic value of an outcome.

However, it is also possible that there is some fundamental difference in the nature of the shift in hedonic value produced by these two devaluation procedures. The effects of satiety involve a temporary reduction in positive hedonic value, whereas taste aversion produces a long lasting positive-to-negative change in hedonic value. It is therefore important to test the extent to which specific PIT is sensitive to changes in the hedonic value of the specific outcomes that do not involve aversion. This could be achieved by either reducing or inflating the hedonic value of a specific outcome immediately before a PIT test using incentive learning/contrast effects (e.g., Experiment 3 and 4 in Balleine and Dickinson, 1998), or by pharmacologically inducing specific nutrient deprivation states relevant to specific outcomes (Krieckhaus and Wolf, 1968; Fudim, 1978; Davidson et al., 1997).

Further experiments are still needed to directly test the role of sensory habituation in specific PIT following specific satiety. One approach would be to test whether the ability for specific satiety to disrupt specific PIT is context specific in the same manner, and over the same long time scales, as reported for instrumental outcome devaluation (Parkes et al., 2016). Another prediction of this habituation account is that it should be disrupted by a dishabituation manipulation. For example, briefly presenting a novel taste immediately after the specific satiety manipulation should recover specific PIT. Similarly, presenting multiple outcomes during outcome devaluation should also disrupt sensory habituation during a specific satiety devaluation. Indeed, Lingawi and colleagues have shown, using a S_1_-O_1_/S_2_-O_2_ and R_1_-O_1_/R_2_-O_2_ specific PIT design, that devaluing both O_1_ and O_2_ during the same specific satiety consumption period attenuates but does not completely abolish specific PIT (Figure 2 in Lingawi et al., 2022).

More generally, the present discussion also highlights the importance of testing multiple aspects of the specific PIT effect. Tests of specific PIT should assess (1) outcome specificity: greater responding on the same than different lever, and (2) a facilitative PIT transfer effect: responding on the same lever above baseline. Consistent statistical reporting of both of these tests would enhance comparisons made between studies, and may reveal important psychological and neural distinctions between the signaling and the response invigorating properties of stimuli in PIT (Delamater and Holland, 2008; Holmes et al., 2010; Laurent and Balleine, 2015; Marshall et al., 2022).

Given the ubiquity of interactions between stimuli and instrumental actions in the daily lives of human and non-human animals, further research is required to improve our current understanding of the nature of PIT effects. Significant progress has been made recently with an increasing interest in PIT research such as, establishing the relevant contents of learning involved in PIT (Gilroy et al., 2014; Laurent and Balleine, 2015; Lingawi et al., 2016; Laurent et al., 2021), identifying the boundary conditions of general PIT effects (Holmes et al., 2010; Ostlund and Marshall, 2021), understanding the role of PIT in substance use (Ostlund and Marshall, 2021), and revealing fine grained neural circuits underlying PIT and their signaling processes (Laurent et al., 2014; Lichtenberg et al., 2017; Bradfield et al., 2018; Sias et al., 2021).

## Data availability statement

Data are available at DOI 10.17605/OSF.IO/NHT6A.

## Ethics statement

The animal study was reviewed and approved by University of New South Wales Animal Care and Ethics Committee.

## Author contributions

MP and SK designed the experiments and wrote the manuscript. MP conducted the experiments and analyzed the data. All authors contributed to the article and approved the submitted version.

## Funding

Research supported by grants awarded to SK from the Australian Research Council (ARC Discovery Grant DP0989027 and DP120103564).

## Conflict of interest

The authors declare that the research was conducted in the absence of any commercial or financial relationships that could be construed as a potential conflict of interest.

## Publisher's note

All claims expressed in this article are solely those of the authors and do not necessarily represent those of their affiliated organizations, or those of the publisher, the editors and the reviewers. Any product that may be evaluated in this article, or claim that may be made by its manufacturer, is not guaranteed or endorsed by the publisher.
