# Supplementary material for: Outcome devaluation by specific satiety disrupts sensory-specific Pavlovian-to-instrumental transfer
Source: Front Behav Neurosci. 2022 Nov 9;16:983480. doi: 10.3389/fnbeh.2022.983480 (PMC9682038; doi:10.3389/fnbeh.2022.983480)
Supplement: Supplementary file 1 [file Data_Sheet_1.PDF]

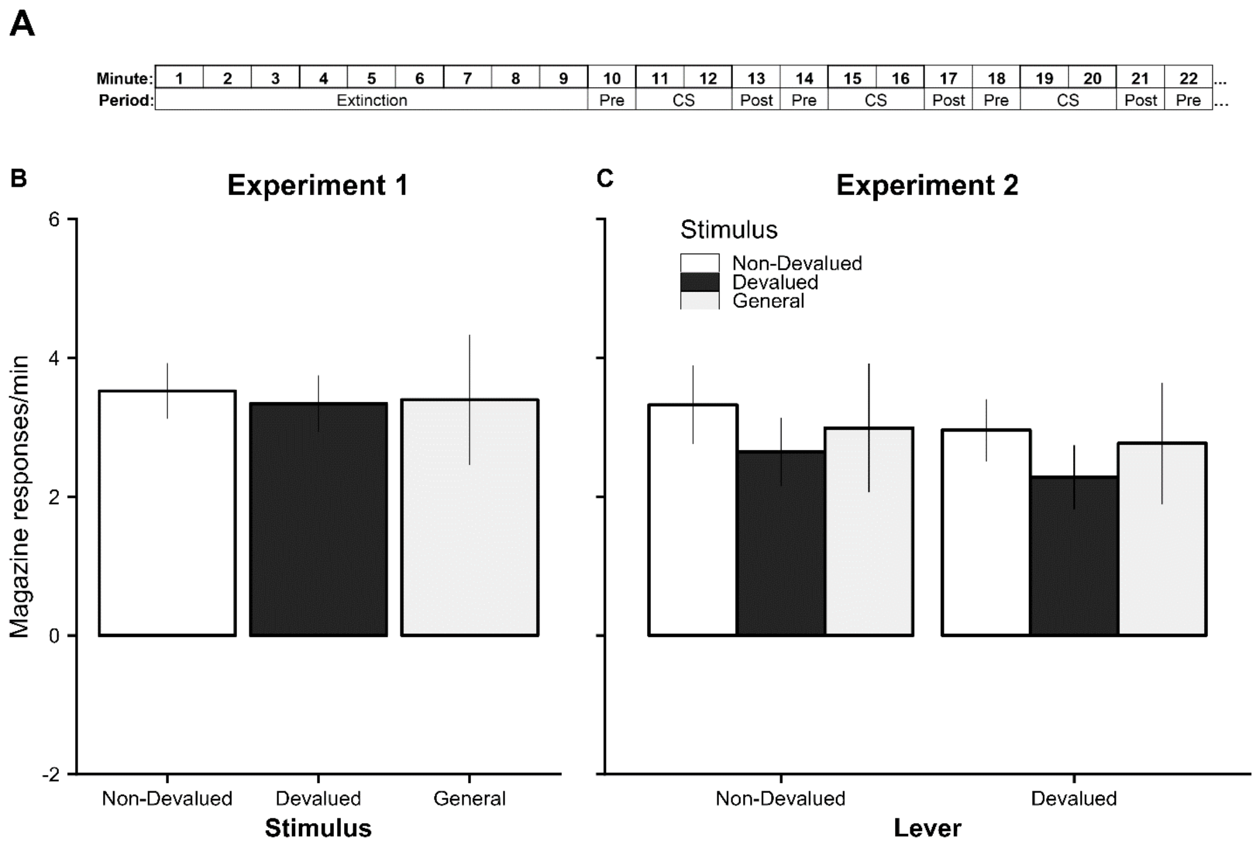

Supplementary Figure 1. Definition of time periods analysed during the PIT tests, and magazine responding at test in each experiment. (A) The structure of the PIT test is depicted with time in minutes on the top, and the corresponding analysis period indicated below. The first 9 minutes of the extinction period were used as the Extinction period in which only the lever(s) were present. After the first 10 minutes the Pavlovian stimuli (CS) were presented for 2 minutes, followed by a two-minute inter-stimulus interval. The baseline (Pre) period was defined as the minute immediately prior to each CS. This baseline period was analysed separately (depicted in Figure 1F and 2F) and then the rate of responding (per minute) was subtracted from the rate of responding (per minute) of the corresponding CS that followed. The 1-minute period immediately following each CS (Post) was not included in the baseline measurement as it contained elevated responding that persisted for a short while after the end of the previous CS. Elevation in the rate of magazine responding (CS-PreCS) in the presence of each cue during the PIT tests in (B) Experiment 1 and (C) Experiment 2. Note that for the single-lever tests performed in Experiment 2, responding is separated by whether the lever that was present during the test predicted a Non-Devalued (C, Left) or Devalued (C, Right) outcome. Data are presented as mean ±SEM.

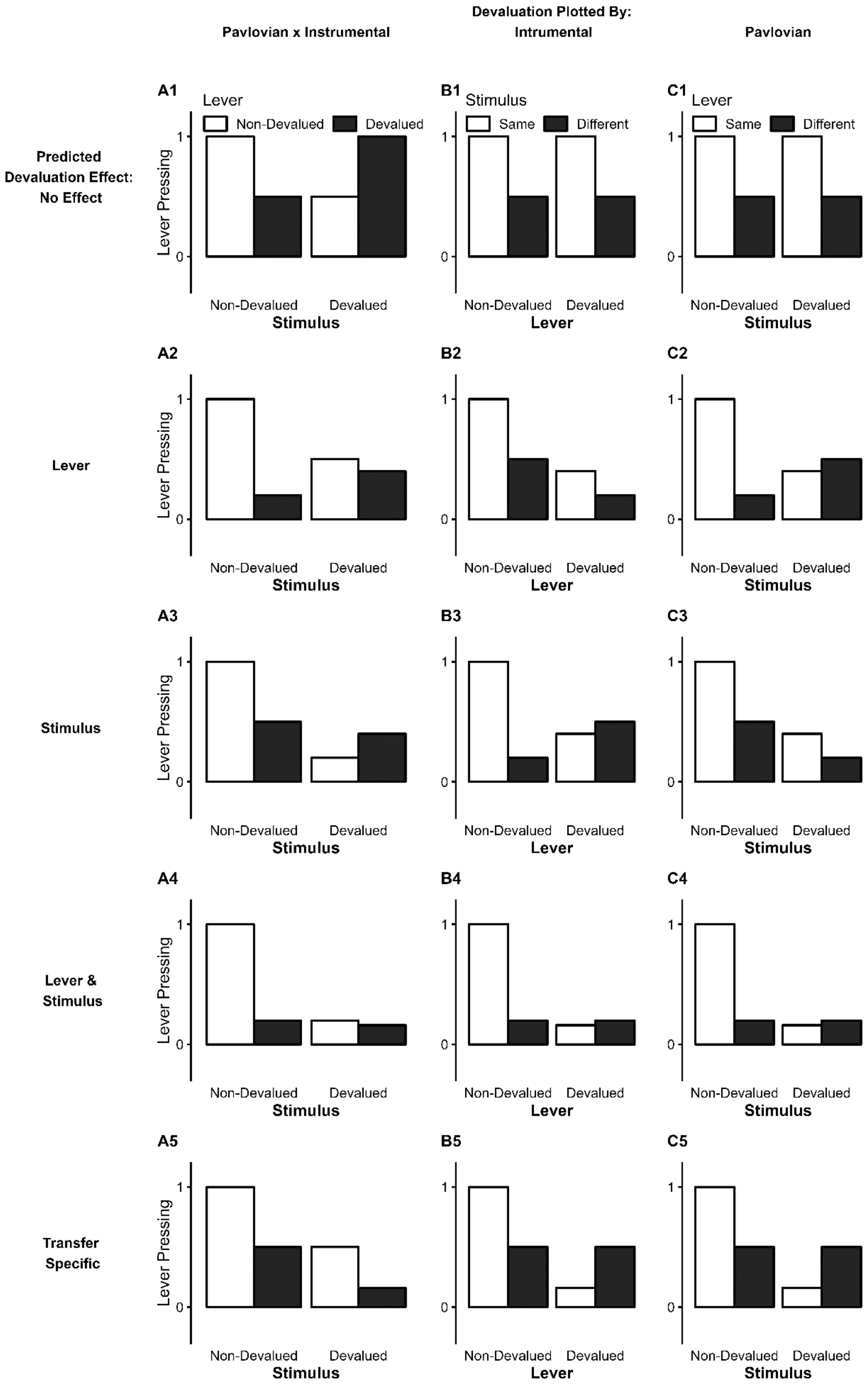
Devaluation Plotted By:
Pavlovian x Instrumental
Intrumental
Pavlovian
Predicted Devaluation Effect:
No Effect
Lever
Stimulus
Lever & Stimulus
Transfer Specific
A1
B1
C1
A2
B2
C2
A3
B3
C3
A4
B4
C4
A5
B5
C5
Lever
Non-Devalued
Devalued
Stimulus
Same
Different
Lever Pressing
1
0
Non-Devalued
Devalued
Stimulus
Lever

Supplementary Figure 2. Multiple approaches to analyzing the effect of outcome devaluation on specific PIT. Specific PIT data are normally plotted to clearly show whether the predicted outcomes of the instrumental lever and the Pavlovian stimuli are the "Same" or "Different". This leads to four possible lever-stimulus combinations Non-Devalued Stimulus:Non-Devalued Lever (Same), Non-Devalued Stimulus:Devalued Lever (Different), Devalued Stimulus:Devalued Lever (Same), Devalued Stimulus:Non-Devalued Lever (Different). Unfortunately, this complexity can sometimes obscure some feature of the underlying effects that are observed depending on how the data are plotted. In this figure, we demonstrate this with some idealized model predicted data that are described in detail in the Supplementary Model Description below. There are three possible methods of plotting specific PIT test data following outcome devaluation (plotted in separate columns: A, B, and C), and each method has some advantages and disadvantages when trying to understand the underlying pattern of data (rows 1-5 depict five different possible effects of outcome devaluation). First we will describe each column, which represents a different method of plotting the same data (for a given row). (A) Pavlovian x Instrumental: Plotting lever pressing separated by whether the Pavlovian stimulus is devalued (x-axis) against the devaluation status of the instrumental lever (separate bars). (B) Instrumental: Plotting lever pressing separated by whether the instrumental lever is devalued (x-axis) while comparing whether the Pavlovian stimulus predicts the same or different outcome (separate bars). (C) Pavlovian: Plotting lever pressing separated by whether the Pavlovian stimulus is devalued (x-axis) while comparing whether the instrumental lever was trained with the same or different outcome (separate bars). Note that, for a given row, the data in plotted in columns A, B, and C are identical i.e., each column represents a different way to plot the same data. In contrast, each row (labeled 1-5) represents a different predicted pattern of results, modeling a range of possible effects of devaluation on specific PIT responding. (1) No Effect: the basic PIT effect i.e., no effect of devaluation on any aspect of the specific PIT effect. Note that it is straight forward to see from plots A2 and A3 that the devaluation has not had any effect on the basic specific PIT effect. In contrast, this conclusion is not as intuitive when looking at the same data in panel A1 where the Same and Different conditions are not clearly indicated. (2) Lever: the predicted impact of a main effect of devaluation that only impacts lever responding i.e., reduction in devalued vs non-devalued lever responding regardless of the Pavlovian stimulus that is present. (3) Stimulus: the predicted impact of a main effect of Stimulus devaluation, but no instrumental devaluation effect i.e., a reduction in responding to the devalued vs non-devalued stimulus. (4) Lever & Stimulus: the predicted impact of independent main effects of both Stimulus devaluation and instrumental devaluation i.e. responding on the devalued lever is lower than the non-devalued lever, and the devalued stimulus is less effective at eliciting lever responding than the non-devalued stimulus. (5) Transfer Specific: devaluation only affects the Same condition i.e., the devalued lever in the presence of the devalued stimulus. These three plotting methods can help reveal whether the impact of devaluation is specific to the Pavlovian stimuli, instrumental levers, or some combination of both. For example, a main effect of instrumental devaluation is easily detected when separating responding by devalued and non-devalued levers (i.e., B2), whereas a main effect of Pavlovian devaluation is easily detected when separating responding by devalued and non-devalued stimuli (i.e., C3). It is notable that studies of outcome devaluation and its effect on specific PIT with single-lever tests have separated the data by instrumental devaluation (i.e. column B) (Colwill & Rescorla, 1990; Holland, 2004; Rescorla, 1994), whereas those with two-lever tests have separated the data by Pavlovian devaluation (i.e. column C) (Lingawi et al., 2022; Sommer et al., 2022).

Supplementary Model Description

To illustrate the impact of different effects of devaluation on responding, plotted in Supplementary Figure 2, we modelled these effects using following formula:

$Y_{Pred} = \beta_{PIT} * (1 + (X_{InstD}*\beta_{InstD})) * (1 + (X_{PavD}*\beta_{PavD}))$,

Predicted Responding = [Basic PIT Effect] x [Instrumental Devaluation] x [Pavlovian Devaluation]

Where: Y_{Pred} = Predicted Responding, Basic PIT Effect = β_{PIT} , both of which are response probabilities. Instrumental Devaluation = $(1 + (X_{InstD}*\beta_{InstD}))$, where $X_{InstD} \in \{0,1\}$ is a dummy variable coding whether to include the effect of instrumental devaluation, and β_{InstD} is the reduction in responding (expressed as a proportion) representing the impact of instrumental devaluation on responding. Pavlovian Devaluation = $(1 + (X_{PavD}*\beta_{PavD}))$ where $X_{PavD} \in \{0,1\}$ is a dummy variable coding whether to include the effect of Pavlovian devaluation, and β_{PavD} is the reduction in responding (expressed as a proportion) representing the impact of Pavlovian devaluation on responding. For Same: $\beta_{PIT} = 1$, and Different: $\beta_{PIT} = 0.5$, reflecting a PIT effect such that responding on the same lever is double responding on the Different lever. These are modeled approximately upon the mean values from Experiment 2, as well as data from non-devalued subjects using similar parameters (Panayi & Killcross, 2018). The magnitude of devaluation for both instrumental (β_{InstD}) and Pavlovian (β_{PavD}) effects was {Non-Devalued, Devalued} = {0, -0.6}, reflecting no impact for a non-devalued condition (0), and a 60% reduction in responding for a devalued condition (-0.6). This was modeled on the average percentage reduction in devalued compared to non-devalued lever pressing during the Extinction tests in Experiment 1 and 2, which range between approximately 50-60% reduction. These parameters are presented in Supplementary Table 1. For example, consider model the impact of a main effect of Instrumental devaluation on the Different condition created by the Devalued lever in the presence of the Non-Devalued Stimulus. The basic PIT effect for the Different condition is $\beta_{PIT} = 0.5$, the lever is devalued ($X_{InstD} = 1$, $\beta_{InstD} = -0.6$) but the Pavlovian stimulus is not devalued ($X_{PavD} = 0$, $\beta_{PavD} = 0.6$). Accordingly, predicted responding would be calculated as $Y_{Pred} = 0.5 * (1 + (1*-0.6)) * (1 + (0*-0.6)) = 0.2$. The complete set of modeled conditions is presented in Supplementary Table 2. Note that this model contains many assumptions about the nature of responding in PIT (e.g., it ignores baseline responding), devaluation (e.g., assumes equivalent impact of instrumental and Pavlovian devaluation on lever responding), and behavioural constraints (e.g. floor and ceiling effects). The specific parameters chosen were also loosely modeled on the available data from the present experiments to aid visual similarity when plotted in Supplementary Figure 2 and were not formally modeled.

Experimental Parameters			Parameters: β		
Lever	Stimulus	Condition	β_{PIT}	β_{InstD}	β_{PavD}
NonDevalued	NonDevalued	Same	1	0	0
NonDevalued	Devalued	Different	0.5	0	-0.6
Devalued	NonDevalued	Different	0.5	-0.6	0
Devalued	Devalued	Same	1	-0.6	-0.6

Supplementary Table 1. *Parameters used in the modeled data plotted in Supplementary Figure 2.* The devaluation status of each Lever and Stimulus creates a corresponding Same or Different condition. For each condition, there is a Basic PIT Effect = β_{PIT}, which reflects the expected magnitude of the Same/Different effect, independent of the devaluation manipulation. The corresponding impact of Instrumental (β_{InstD}) and Pavlovian (β_{PavD}) devaluation for each condition represents a reduction of 60% for a devalued condition or 0% for a non-devalued condition.

Predicted Devaluation	Experimental Parameters			Effect Coding		
	Lever	Stimulus	Condition	X_{InstD}	X_{PavD}	Y_{Pred}
No Effect	NonDevalued	NonDevalued	Same	0	0	1
	NonDevalued	Devalued	Different	0	0	0.5
	Devalued	NonDevalued	Different	0	0	0.5
	Devalued	Devalued	Same	0	0	1
Lever	NonDevalued	NonDevalued	Same	1	0	1
	NonDevalued	Devalued	Different	1	0	0.5
	Devalued	NonDevalued	Different	1	0	0.2
	Devalued	Devalued	Same	1	0	0.4
Stimulus	NonDevalued	NonDevalued	Same	0	1	1
	NonDevalued	Devalued	Different	0	1	0.2
	Devalued	NonDevalued	Different	0	1	0.5
	Devalued	Devalued	Same	0	1	0.4
Lever & Stimulus	NonDevalued	NonDevalued	Same	1	1	1
	NonDevalued	Devalued	Different	1	1	0.2
	Devalued	NonDevalued	Different	1	1	0.2
	Devalued	Devalued	Same	1	1	0.16
Transfer Specific	NonDevalued	NonDevalued	Same	1	1	1
	NonDevalued	Devalued	Different	0	0	0.5
	Devalued	NonDevalued	Different	0	0	0.5
	Devalued	Devalued	Same	1	1	0.16

Supplementary Table 2. *Parameters used for each condition in the modeled data plotted in Supplementary Figure 2.* X_{InstD} and X_{PavD} code for whether the impact of Pavlovian or instrumental devaluation is being modeled for each of the Predicted Devaluation effects. Predicted responding (Y_{Pred}) is calculated in conjunction with the values from Supplementary Table 1, using the following formula: $Y_{Pred} = \beta_{PIT} * (1 + (X_{InstD}*\beta_{InstD})) * (1 + (X_{PavD}*\beta_{PavD}))$. See Supplementary Model Description for details.

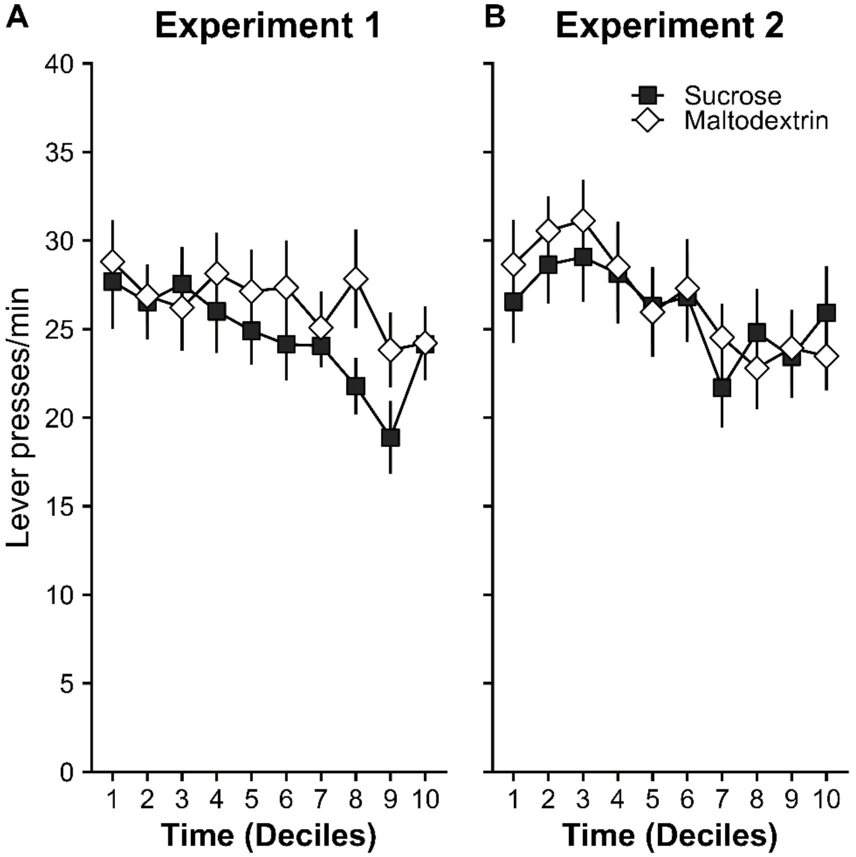

Supplementary Figure 3. Evidence of within-session decline in rates of instrumental responding in Experiment 1 and 2. Rates of within-session lever pressing during the final two days of instrumental training (days 5 and 6) on an RR10 schedule during both experiments. The duration of each session was dependent on individual rates of responding, but on average lasted 10 minutes. The total time within each session was split into 10 epochs of equal duration (i.e., deciles) to allow for an analysis of changes in responding over time. (A) Experiment 1. Responding for both sucrose and maltodextrin outcomes significantly decreased within-session (main effect of Deciles, $F(9{,}135) = 2.54$, $p = .010$; significant negative linear trend over Deciles, $t(15) = -3.59$, $p = .003$), and at comparable rates for both rewards (no main effect of Reward, ($F(1{,}15) = 1.56$, $p = .231$), or Decile*Reward interaction, $F(9{,}135) = 1.04$, $p = .414$). (B) Experiment 2. Responding for both sucrose and maltodextrin outcomes significantly decreased within-session (main effect of Deciles, $F(9{,}117) = 3.25$, $p = .001$; significant negative linear trend over Deciles, $t(13) = -4.09$, $p = .001$), and at comparable rates for both rewards (no main effect of Reward, ($F(1{,}13) = 0.00$, $p = .994$), or Decile*Reward interaction, $F(9{,}117) = 1.18$, $p = .312$). Data are presented as mean ±SEM. It is noteworthy that the present experiments were not optimally designed for producing a within-session decline and did not contain any method of assessing whether they were driven by habituation processes (Epstein, Temple and Bouton, 2009; McSweeney and Murphy, 2009; Bouton et al, 2013). The sessions were short and variable (10 mins on average), with relatively few reinforcers (20), and employed a random schedule of reinforcement (RR10). A more optimal design for producing a within-session decline driven by habituation would prioritize longer sessions (30-60 mins) with a more predictable and consistent schedule of reinforcement (fixed ratio or fixed interval schedules).
